# Supplementary material for: Temporal evolution of master regulator Crp identifies pyrimidines as catabolite modulator factors
Source: Nat Commun. 2021 Oct 7;12:5880. doi: 10.1038/s41467-021-26098-x (PMC8497467; doi:10.1038/s41467-021-26098-x)
Supplement: Supplementary file 1 — Supplementary Information [file 41467_2021_26098_MOESM1_ESM.pdf]

Supplementary information for:

Temporal Evolution of Master Regulator Crp Identifies Pyrimidines as Catabolite Modulator Factors

Ida Lauritsen[1,3], Pernille Ott Frendorf[1,3], Silvia Capucci[1], Sophia E. H. Heyde[1], Sarah D. Blomquist[1], Sofie Wendel[1], Emil C. Fischer[1], Agnieszka Sekowska[2], Antoine Danchin[2] and Morten H. H. Nørholm[1,4]

[1]Novo Nordisk Foundation Center for Biosustainability, Technical University of Denmark, Kemitorvet B220, DK-2800 Kgs. Lyngby, Denmark

[2] Kodikos Labs, Institut Cochin, 24 rue du Faubourg Saint Jacques, 75014 Paris, France

[3]These authors contributed equally

[4]Correspondance to morno@biosustain.dtu.dk

Supplementary Table 1– Crp mutants identified in 594 sequenced *crp* loci from ageing bacteria[1]. All selected colonies exhibited an papillae phenotype on MacConkey agar supplied with maltose. 71 clones out of 594 did not contain a *crp* mutation.

**CRP mutation 1**	**type**	**CRP paired mutation**	**type**	**count**
A144T	G->A			307
A144T	G->A	L11I	C->A	1
A144T	G->A	I30F	A->T	8
A144T	G->A	L39M	C->A	2
A144T	G->A	D53N	G->A	1
A144T	G->A	E55K	G->A	1
A144T	G->A	E55D	G->T	1
A144T	G->A	E55E	G->A	1
A144T	G->A	S62F	C->T	3
A144T	G->A	S62Y	C->A	1
A144T	G->A	L64Q	T->A	1
A144T	G->A	R82S	C->A	1
A144T	G->A	A84E	C->A	2
A144T	G->A	W85R	T->A	1
A144T	G->A	P110Q	C->A	1
A144T	G->A	T127I	C->T	2
A144T	G->A	N133H	A->C	1
A144T	G->A	T140K	C->A	2
A144T	G->A	Q145K	C->A	1
A144T	G->A	T146A	A->G	1
A144T	G->A	D155N	G->A	1
A144T	G->A	Q170K	C->A	5
A144T	G->A	M189K	T->A	1
A144T	G->A	M189I	G->A	1
A144T	G->A	Q193K	C->A	1
A144T	G->A	3 aa insertion		1

A144E	C->A			57
A144E	C->A	I30F	A->T	2
A144E	C->A	L39M	G->A	3
A144E	C->A	E55K	C->A	1
A144E	C->A	S62Y	C->A	4
A144E	C->A	A84E	C->A	1
A144E	C->A	Q119H	G->T	1
A144E	C->A	T140K	C->A	7
A144E	C->A	Q170K	C->A	7
A144E	C->A	Q174K	T->A	2
A144E	C->A	M189K	T->A	4
A144E	C->A	22 aa insertion		1
T140K	C->A			7
T140K	C->A	M189R	T->G	1
T140K	C->A	insertion of N between G56 and K57		1
T140R	C->G	T140R		16
T140R	C->G	Q119H	C->G	1
T140R	C->G	Q170K	C->G	1
T140R	C->G	V183A	T->C	1
M189K	T->A			3
M189K	T->A	Y63F	A->T	1
M189K	T->A	Q170K	C->A	1
insertion K57				11
insertion K57		A84E	C->A	2

S62Y	C->A		1
P110Q	C->A		2
L134M	C->A		1
T140P	A->C		1
G141S	G->A		1
G141D	G->A		9
A144K	G->A and C->A		12
L195R	T->G		4
insertion of N between G56 and K57			1
insertion of L between V86 and R87			1
insertion of V between V86 and R87			1
duplication 3 aa 86-88			1
duplication of 20 aa			2
duplication of 28 aa			1
No mutation			71
		SUM	594

Supplementary Table 2– *crp* mutations detected in *crp* loci from papillae.

**Background strain**	**Plasmid**	**Condition**	**CRP mutation(s)**
cya crp	pSEVA27-*crp*	maltose	
papillae 1			A144T
papillae 2			A144T, R82L
papillae 3			A144T, M198I
papillae 4			A144E
papillae 5			A144T, A135V
papillae 6			A144E
cya crp cmk	pSEVA27-*crp*	maltose	
papillae 1			A144E
papillae 2			130I, T140K
papillae 3			D53Y
papillae 4			A144T
papillae 5			A144E
papillae 6			A144T
cya crp cmkA216E	pSEVA27-*crp*	maltose	
papillae 1			A144T
papillae 2			A144T
papillae 3			A144T
papillae 4			A144T
papillae 5			A144T
papillae 6			A144T
cya	none		
papillae 1		maltose+cytidine	A144T
papillae 2		maltose+cytidine	A144T
papillae 3		maltose+cytidine	A144T
papillae 4		maltose+cytidine	A144T
papillae 5		maltose+cytidine	A144T
papillae 6		maltose	R122H, duplication K52

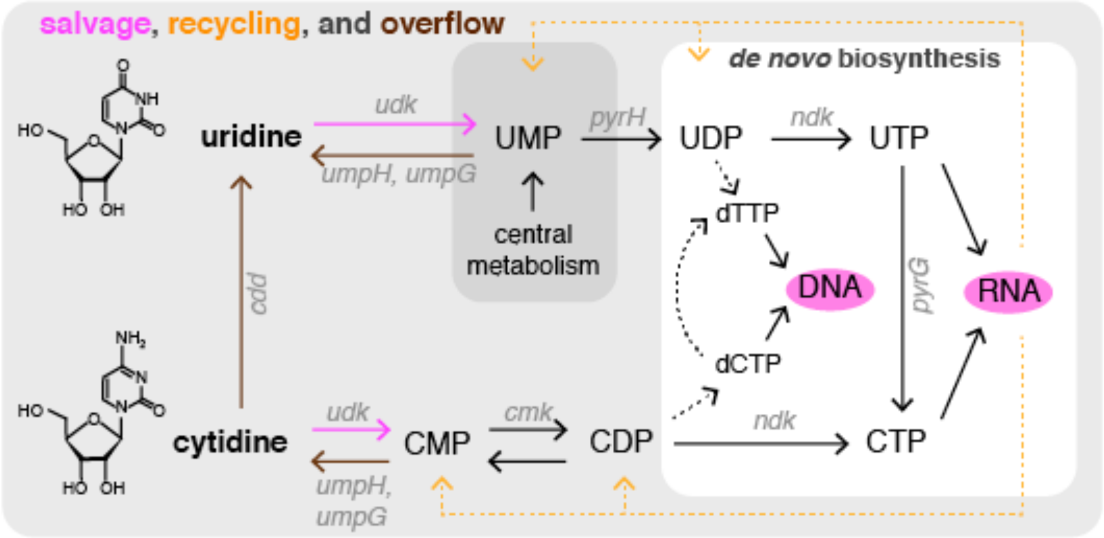

Supplementary Fig. 1. Pyrimidine metabolism. Pyrimidines materialize in living cells by *de novo* synthesis from central metabolites, salvage via uptake from the environment, recycling mainly from RNA, or directed overflow from nucleotides.

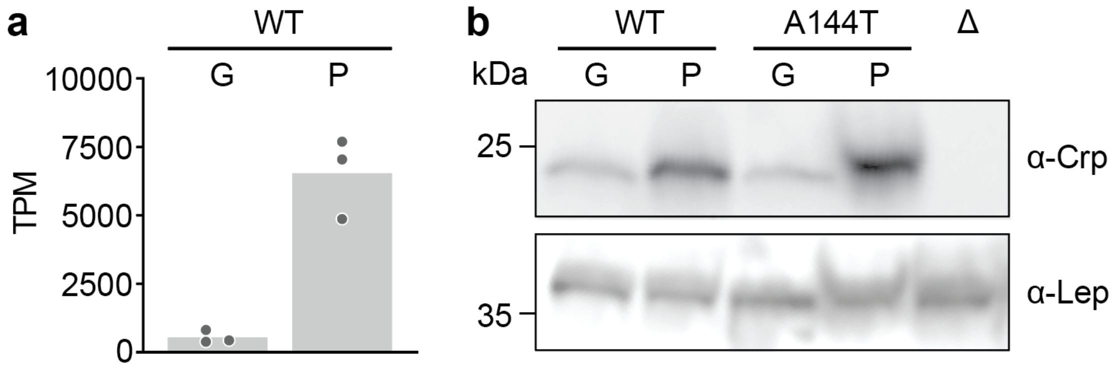

Supplementary Fig. 2 - Crp expression increases in Δ*cyaA* strains when expressed from a plasmid (Δ*crp* pSEVA27-P*crp*, P) compared to the genome (native context, G). a) *crp* (WT) mRNA levels (transcripts per million, TPM) determined by transcriptomics. Data represent the average of three biological replicates. Significant (p=0.0023) based on a two-sided unpaired t-tests between the groups. b) Western blot of Crp (WT or A144T) compared to a Δ*crp* strain (above) with an internal control for protein levels (Lep, below). α designates the protein-specific antibodies applied. Data represent the observed results from two biological replicate experiments.

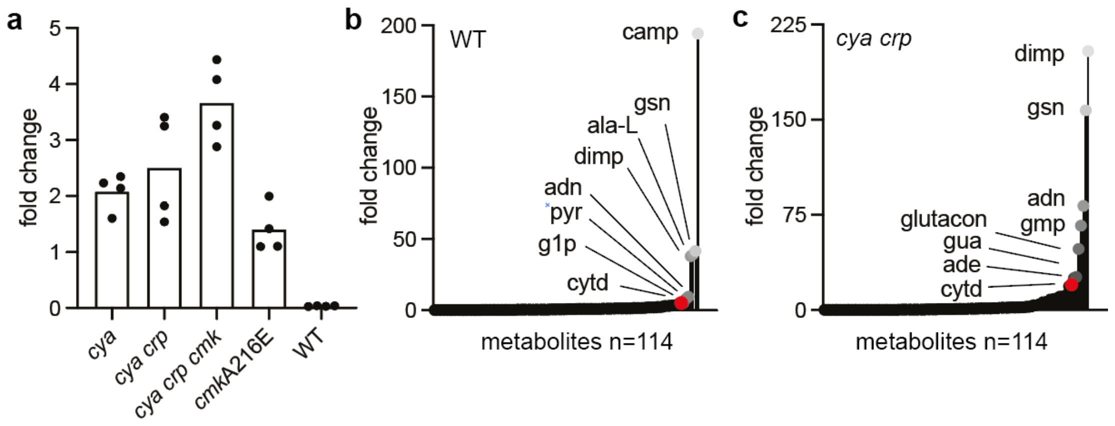

Supplementary Fig. 3 – Cytidine accumulates during ageing of bacterial strains. a) Fold change of absolute CMP levels in for day 1 to 5. Data represent four replicates. b) Distribution of fold changes of all metabolites detected by LC-MS from day 1 to 6 for WT. Metabolites with the highest fold change are highlighted. camp: cyclic adenosine monophosphate, gsn: guanosine, ala-L: alanine, dimp: deoxyinosine monophosphate, adn: adenosine, pyr: pyruvate, g1p: glucose-1-phosphate, cytd: cytidine. c) Distribution of fold changes of all metabolites detected by LC-MS from day 1 to 6 for *cya crp*. Metabolites with highest fold change are highlighted. Light to dark grey: dimp: deoxyinosine monophosphate, gsn: guanosine, adn: adenosine, gmp: guanosine monophosphate, glutacon: glutaconate, gua: guanine, ade: adenine, cytd: cytidine (red). Data represents the average of four replicates.

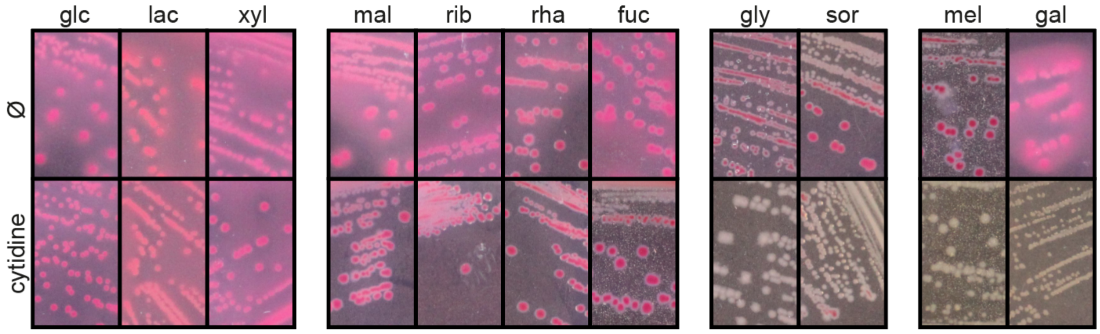

Supplementary Fig. 4 – Phenotypes of MG1655 on MacConkey agar supplemented with designated carbon sources. Phenotypes are grouped by patterns of fermentation when supplemented further with 10 mM cytidine (cyt). Grouped (left to right) by no effect, decreased media acidification, decreased fermentation, and no fermentation. glc: glucose, lac: lactose, xyl: xylose, mal: maltose, rib: ribose, rha: rhamnose, fuc: fucose, gly: glycerol, sor: sorbitol, mel: melibiose, gal: galactose.

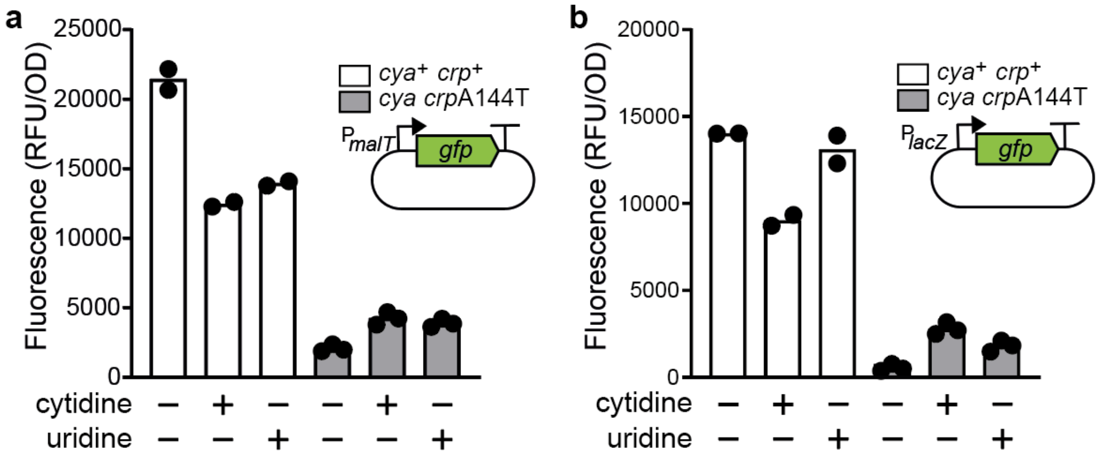

Supplementary Fig. 5 – *In vivo* Crp activity measured with different CRP responsive reporters in the presence of the nucleosides cytidine or uridine. a, Crp activity reporter with the P*malT* promoter controlling expression of GFP, or **b**, P*lacZ*. Crp activity was measured in a wildtype *crp* (white) or *crp*A144T (grey) strain background after nine hours of growth. Data represent two or three independent clones for each condition.

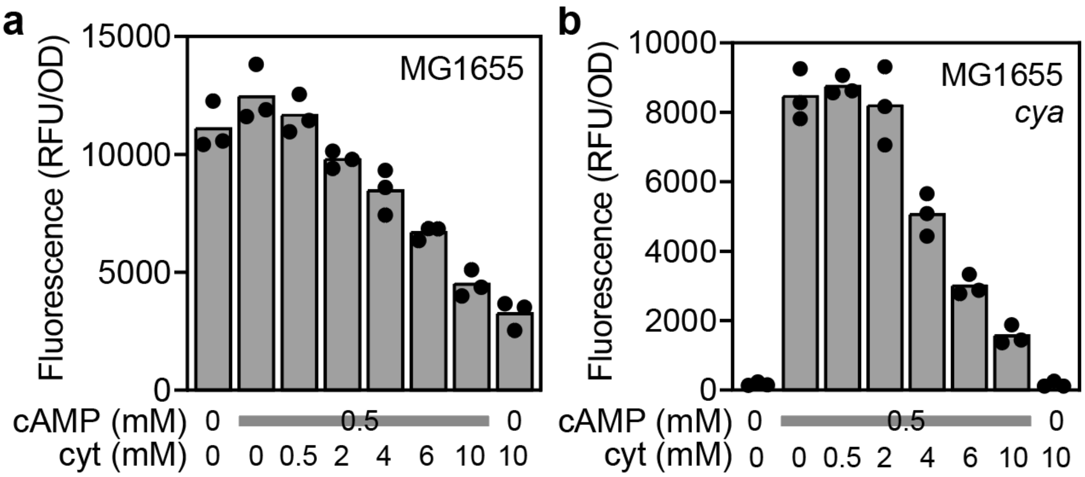

Supplementary Figure 6. Cytidine titration during cAMP supplementation of the pGEM-P*malT* reporter. a) MG1655 at 9 h of growth. b) MG1655 *cya* at 9 h of growth. Data represents biological triplicates.

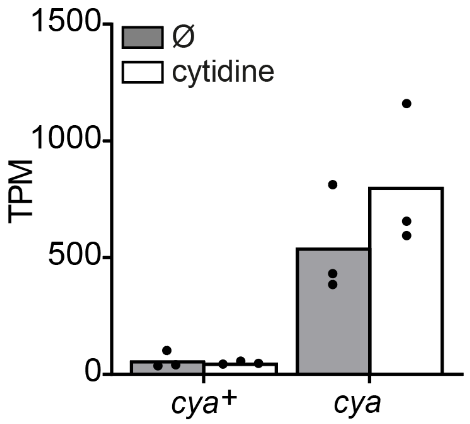

Supplementary Fig. 7. *crp* mRNA levels (TPM, transcripts per million) as determined by transcriptomics for MG1655 (*cya*$^{+}$) and its *cya* derivative when supplemented with no (Ø, grey) or 10 mM cytidine (white). Significance was based on two-sided unpaired t-tests between the groups, and designated as significant if $p<0.05$. Data represent the average of three biological replicates.

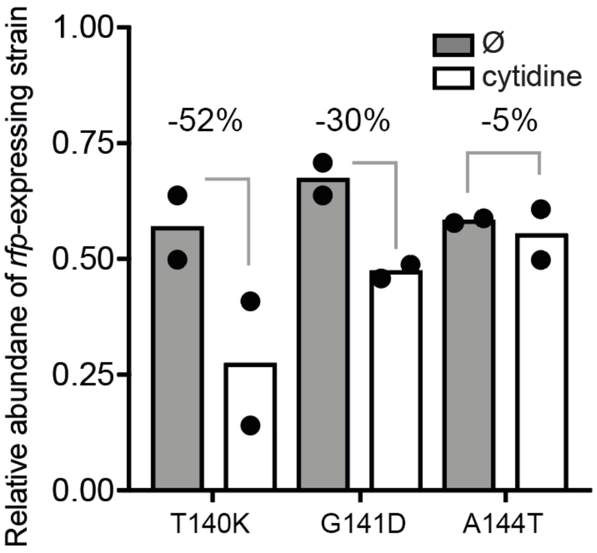

Supplementary Fig. 8 - Competition assays between CrpA144T and CrpT140K or G141D. Relative abundance of strains carrying the Crp* mutations T140K and G141D in competition with A144T when grown on agar plates with maltose. The two competing strains, expressing either *gfp* or *rfp,* were mixed in equal proportions and a 10 µl drop of 0.5 mM cytidine (white) or water (Ø, grey) was applied to the center of the agar plate for diffusion. RFP fluorescence measurements of cells taken from different sections of the agar plate were used to determine the cytidine diffusion gradient. The relative abundance was calculated based on the fluorescence levels for one strain expressing *rfp*. Data represent the average of two biological replicates with standard deviations. The reporter plasmids expressing either *rfp* or *gfp* of the competing strains are switched compared to data presented in Fig. 6e. The data for A144T in competition with A144T is the same as displayed in Fig. 6e.

Supplementary Table 3. Strains and plasmid used in this study

Strains	Description/relevant characteristics	Reference
WT	MG1655 K-12 WT	Our laboratory
cya	K-12 MG1655 *cyaA::cat* Δ*fnr*	[1]
crp	K-12 MG1655 *cyaA::cat* Δ*fnr* Δ*crp*	This study
*crp*A144T	MG1655 K-12 *cyaA::cat* Δ*fnr* - *crp*A144T	[1]
*cmk*A216E	MG1655 K-12 *cyaA::cat* Δ*fnr* Δ*crp* *cmk*A216E	This study
*cmk*A216E-*crp*	*MG1655 K-12 cyaA::cat Δfnr cmk*A216E	[1]
*crp*A144T-Q170K	MG1655 K-12 *cyaA::cat* Δ*fnr* *crp*A144T-Q170K	[1]
BL21(DE3)	F– *omp*T *hsdS*$_B$ (r_B–, m_B–) *gal dcm* (DE3)	ThermoFischer Scientific
crp cmk	K-12 MG1655 *cyaA::cat* Δ*fnr* Δ*crp* Δ*cmk*	This study
crp cytR	K-12 MG1655 *cyaA::cat* Δ*fnr* Δ*crp* Δ*cytR*	This study
crp rpoH	K-12 MG1655 *cyaA::cat* Δ*fnr* Δ*crp* Δ*rpoH*	This study

Plasmids	Description/relevant characteristics	Reference
pSEVA27	pSC101 replicon, Kan^R	[2]
pCDF-*crp*	*crp* expression from *trc* promoter, CloDF13 replicon, Sp^R	Our laboratory
pCDF-*crp*A144T	*crp*A144T expression from *trc* promoter, CloDF13 replicon, Sp^R	Our laboratory
pSEVA-Ptrc-*crp*A144T	*crp*A144T*,* expression from *trc* promoter, pSC101 replicon, Kan^R	This study
pSEVA27-*crp*	*crp* expression from native *crp* promoter, pSC101 replicon, Kan^R	This study
pSEVA27-*crp*A144T	*crp*A144T expression from native *crp* promoter, pSC101 replicon, Kan^R	This study
pSEVA27-*crp*T140K	*crp*T140K expression from native *crp* promoter, pSC101 replicon, Kan^R	This study
pSEVA27-*crp*G141D	*crp*G141D expression from native *crp* promoter, pSC101 replicon, Kan^R	This study
pZE21-sfGFP-Kan	*sfgfp* expression from *tet* promoter, constitutively (no *tetR),* ColE1 replicon Kan^R	Dr. Andreas Porse
pZE21-RFP-Kan	*rfp* expression from *tet* promoter, constitutively (no *tetR),* ColE1 replicon, Kan^R	Dr. Andreas Porse

pZE21-sfGFP	*sfgfp* expression from *tet* promoter, constitutively (no *tetR),* ColE1 replicon Amp^R	This study
pZE21-RFP	*rfp* expression from *tet* promoter, constitutively (no *tetR),* ColE1 replicon, Amp^R	This study
pSIM19	*beta, exo, gam* expression, pSC101 replicon *repA*^{ts} temperature sensitive, Spec^R	[3]
pET52-*crp*	*crp* expression from T7 promoter, ColE1 replicon, Amp^R	This study
pET52-*crp*A144T	*crp*A144T expression from T7 promoter, ColE1 replicon, Amp^R	This study
pGEM-P*lacZ*-hp-sfGFP	*sfgfp-ssrA*-pHP14 hairpin, expression from *lac*Z promoter, strong SD, ColE1 replicon, Amp^R,	This study<br>This study[3]
pGEM-P*malT*-hp-sfGFP_noKan	*sfgfp-ssrA*-pHP14 hairpin, expression from *malT* promoter, strong SD, ColE1 replicon, Amp^R	This study
pGEM-P*malT*(papillae)-hp-sfGFP_noKan	*sfgfp-ssrA*-pHP14 hairpin, expression from modified *malT* promoter, strong SD, ColE1 replicon, Amp^R	This study
pGEM-P*rpoH*-hp-sfGFP_noKan	*sfgfp-ssrA*-pHP14 hairpin, expression from truncated *rpoH* promoter, strong SD, ColE1 replicon, Amp^R	This study

Supplementary Table 4 - Oligonucleotides used in this study

Oligo ID	Name	Sequence (5'→3')
3838	lacI_crp_fw	AAGACTAGUTCACTGCCCGCTTTCCAG
3839	lacI_crp_rev	ATGCCTTUTTAACGAGTGCCGTAAACGACG
1267	pSEVA33-Rev	AAAGGCAUCAAATAAAACGAAAGGCTC
1270	pSEVA33-fwd	ACTAGTCTUGGACTCCTGTTGATAGATC
2697	pET52-rev	ATGGTATAUCTCCTTCTTAAAGTTAAACAAAATTATTTC
2698	pET52-his_fwd	ACCATCAUCACCATCACCACCAC
2710	Crp_fwd	AGATATACCAUGGTGCTTGGCAAACCGCAAAC
2711	Crp_rev	ATGATGGUGACGAGTGCCGTAAACGACGATG
3915	malT_cds	5'Biotin-GTTGTTAATAAAGATTTGGAATTGTGACACAGTGCAAATTCAGACACATAAAAAAACGTC
3916	malT_ts	GACGTTTTTTTATGTGTCTGAATTTGCACTGTGTCACAATTCCAAATCTTTATTAACAAC
4117	pSEVA_Pcrp_fw	ATGGTGCTUGGCAAACCG
4118	pSEVA_Pcrp_rev	AGTCGTATTAAUTTCCTAATGCAGGAGTC
4119	Pcrp_pSEVA_fw	ATTAATACGACUTTTGCTACTCCACTGCGTCAATTTTC
4120	Pcrp_pSEVA_rev	AAGCACCAUGCGCGGTTATC
4255	pGEM_PmalT_fw	AATTGGGATUAGGCAGGGAGGAGTTG
4256	pGEM_PmalT_rv	AATCCCAATUCACTGGCCGTCGTTTTAC
4257	PmalT-php14_rv	AATCTCACUCGAGAGTCGACGTATTAATGGTTATAAGGTCGGCCAGAAAC
4258	php14-RBS-sfGFP_fw	AGTGAGATUGTTGACGGTACCGTATTTTCCTCTAGAAATAATTTTGTTTAACTTTAAGAAGGAGATATACCATGAGCAAAGGCGAAGAGCTGTTCACTG
4516	pZE21_bb_fw	AAACGAUCCTCATCCTGTCTCTTGATC
4517	pZE21-bb_rev	AGCGGGACUCTGGGGTTC
4529	pGEM-PlacZ-fw	AATTGGGATUGCGCAACGCAATTAATGTGAG
4530	pGEM-PlacZ-rv	AATCTCACUCGAGAGTCGACGTTTCCTGTGTGAAATTGTTATCCGCTC
4518	AmpR_pZE21_fw	AGTCCCGCUTTACCAATGCTTAATCAGTGAGGCAC
4519	AmpR_pZE21_rev	AGGATCGTTUATGAGTATTCAACATTTCCGTGTCGC

3734	TetA_int_crp_fw	ACGAGTGCCGTAAACGACGATGGTTTTACCGTGTGCG<br>GAGATCAGGTTCTGATCTTCTGAAAAAATTTATTTGCT<br>TATTAATCATCCGGCTC
3735	TetA_int_crp_rv	ATGGTGCTTGGCAAACCGCAAACAGACCCGACTCTCG<br>AATGGTTCTTGTCTCATTGCCACCAAACATTAATAACG<br>AAGAGATGACAG
4010	TetA_int_cmk_fw	TGCGAGAGCCAATTTCTGGCGCGCGTATTGTAGCGCTT<br>TTTCAATCACTTTGAAAAAATTTATTTGCTTATTAATC<br>ATCCGGCTC
4011	TetA_int_cmk_rev	ATGACGGCAATTGCCCCGGTTATTACCATTGATGGCCC<br>AAGCGGTGCAGGCAAACATTAATAACGAAGAGATGAC<br>AG
4725	USER_rpoH_fw	AATTGGGATUTAAAAGCGTGTTATACTCTTTCCCTGCA<br>A
4726	USER_rpoH_rv	AATCTCACUCGAGAGTCGACGTTCAAATCCTCTCAATC<br>GATATCTTCTGGC
5249	cytRKO_tetAinsul_fw	TTAAGGTAACGCGCGTGTTGATCCCCGGATGATAAGTT<br>CGCAGTCCATTCATGGCATGGATGAGCTCTACAAA
5250	cytRKO_tetAinsul_rev	GTGAAAGCGAAGAAGCAGGAAACTGCCGCGACCATG<br>AAAGACGTTGCCCTCGTTGATATTCAGTCAATTACAAA<br>CATTAATAACGAAGA
5290	rpoHKO_tetAinsul_fw	TTACGCTTCAATGGCAGCACGCAATTTTTTCATCGCGT<br>TCTTTTCCAGCTCATGGCATGGATGAGCTCTACAAA
5291	rpoHKO_tetAinsul_rev	TGACTGACAAAATGCAAAGTTTAGCTTTAGCCCCAGTT<br>GGCAACCTGGATGTTGATATTCAGTCAATTACAAACAT<br>TAATAACGAAGA
5601	PmalT_-35_variants_fw	ATTAATACGUCGACTCTCGAGTGAGATTGTTG
5738	PmalT_papvariant	ACGTATTAAUGGTTATAAGGTAGGCCAGAAACC

Supplementary Table 5 – Promoter sequences applied in pGEM reporter plasmids in Supplementary Table 3.

Promoter	Sequence	Notes promoter layout
malT	GGATTAGGCAGGGAGGAGTTGCGGGGATGAGCAA<br>GGAAATGTGATCTCAACCACTTAAAGCTAGTGCA<br>AACCACAGGATTAGCATCAAATCAATGCAATACA<br>GCGCAGAAAATCTGTATCTAAGTGCAAAAAATGG<br>CCGTTGCGTATTTTCAAAAAGCGGAAGGTAACTC<br>TATAAATTAAGTAAAGGAGTGAAACAGTCTCATA<br>AGTAAAATATCCAGTGTGCTCCATCTCATTCTTA<br>ATAGATTTATTAAGATCATCTTTTTAGATGGCAC<br>TTTCATCAGGAATGAAGAGGAAACCCTTGCTTAA<br>ATGAATCTGATGAACATAAGGGAAACCAGTATTC<br>ACGCTGGATCAGCGTCGTTTTAGGTGAGTTGTTA<br>ATAAAGATTTGGAATTGTGACACAGTGCAAATTC<br>AGACACATAAAAAAACGTCATCGCTTGCATTAGA<br>AAGGTTTCTGGCCGACCTTATAACCATTAAT	Mlc site excluded.
malT(papillae)	GGATTAGGCAGGGAGGAGTTGCGGGGATGAGCAA<br>GGAAATGTGATCTCAACCACTTAAAGCTAGTGCA<br>AACCACAGGATTAGCATCAAATCAATGCAATACA<br>GCGCAGAAAATCTGTATCTAAGTGCAAAAAATGG<br>CCGTTGCGTATTTTCAAAAAGCGGAAGGTAACTC<br>TATAAATTAAGTAAAGGAGTGAAACAGTCTCATA<br>AGTAAAATATCCAGTGTGCTCCATCTCATTCTTA<br>ATAGATTTATTAAGATCATCTTTTTAGATGGCAC<br>TTTCATCAGGAATGAAGAGGAAACCCTTGCTTAA<br>ATGAATCTGATGAACATAAGGGAAACCAGTATTC<br>ACGCTGGATCAGCGTCGTTTTAGGTGAGTTGTTA<br>ATAAAGATTTGGAATTGTGACACAGTGCAAATTC<br>AGACACATAAAAAAACGTCATCGCTTGCATTAGA<br>AAGGTTTCTGGCCTACCTTATAACCATTAAT	Mlc site excluded.<br><br>Point mutation underlined.
rpoH	TAAAAGCGTGTTATACTCTTTCCCTGCAATGGGT<br>TCCGTAGCAGGGAAAGAGACCCCGTTGTCTCTTC<br>CCGGTATTTCATCTCTATGTCACATTTTGTGCGT<br>AATTTATTCACAAGCTTGCATTGAACTTGTGGAT<br>AAAATCACGGTCTGATAAAACAGTGAATGATAAC<br>CTCGTTGCTCTTAAGCTCTGGCACAGTTGTTGCT<br>ACCACTGAAGCGCCAGAAGATATCGATTGAGAGG<br>ATTTGAA	P1 excluded
lacZ	GCGCAACGCAATTAATGTGAGTTAGCTCACTCAT<br>TAGGCACCCCAGGCTTTACACTTTATGCTTCCGG<br>CTCGTATGTTGTGTGGAATTGTGAGCGGATAACA<br>ATTTCACACAGGAA	

References

1. Sekowska, A., Wendel, S., Fischer, E. C., Nørholm, M. H. H. & Danchin, A. Generation of mutation hotspots in ageing bacterial colonies. *Sci. Rep.* **6**, 2 (2016).
2. Silva-Rocha, R. *et al.* The Standard European Vector Architecture (SEVA): A coherent platform for the analysis and deployment of complex prokaryotic phenotypes. *Nucleic Acids Res.* **41**, 666–675 (2013).
3. Datta, S., Costantino, N. & Court, D. L. A set of recombineering plasmids for gram-negative bacteria. *Gene* **379**, 109–115 (2006).
