# Supplementary material for: Temporal evolution of master regulator Crp identifies pyrimidines as catabolite modulator factors
Source: Nat Commun. 2021 Oct 7;12:5880. doi: 10.1038/s41467-021-26098-x (PMC8497467; doi:10.1038/s41467-021-26098-x)
Supplement: Supplementary file 3 — Description of Additional Supplementary Files [file 41467_2021_26098_MOESM3_ESM.pdf]

Description of Additional Supplementary Files

File Name: Supplementary Movie 1
Description: Time lapse movie of papillae formation. A cya crpA144E strain was plated on MacConkey supplied with maltose. After initial colony formation, pictures of two single colonies were taken from the bottom of the plate every 6 min. over a period of five days.

File Name: Supplementary Data 1
Description: Absolute metabolite levels detected with LC-MS from ageing colonies on agar plates and from liquid cultures over time. Fold changes in metabolite levels from day 1 to 6 for cells growing in liquid cultures.
