# Supplementary material for: Temporal evolution of master regulator Crp identifies pyrimidines as catabolite modulator factors
Source: Nat Commun. 2021 Oct 7;12:5880. doi: 10.1038/s41467-021-26098-x (PMC8497467; doi:10.1038/s41467-021-26098-x)
Supplement: Supplementary file 6 — Reporting Summary [file 41467_2021_26098_MOESM6_ESM.pdf]

nature portfolio

Corresponding author(s): Morten H. H. Nørholm

Last updated by author(s): Aug 18, 2021

Reporting Summary

Nature Portfolio wishes to improve the reproducibility of the work that we publish. This form provides structure for consistency and transparency in reporting. For further information on Nature Portfolio policies, see our Editorial Policies and the Editorial Policy Checklist.

Statistics

For all statistical analyses, confirm that the following items are present in the figure legend, table legend, main text, or Methods section.

n/a	Confirmed	
☐	☒	The exact sample size (n) for each experimental group/condition, given as a discrete number and unit of measurement
☐	☒	A statement on whether measurements were taken from distinct samples or whether the same sample was measured repeatedly
☐	☒	The statistical test(s) used AND whether they are one- or two-sided<br>*Only common tests should be described solely by name; describe more complex techniques in the Methods section.*
☐	☒	A description of all covariates tested
☐	☒	A description of any assumptions or corrections, such as tests of normality and adjustment for multiple comparisons
☐	☒	A full description of the statistical parameters including central tendency (e.g. means) or other basic estimates (e.g. regression coefficient) AND variation (e.g. standard deviation) or associated estimates of uncertainty (e.g. confidence intervals)
☐	☒	For null hypothesis testing, the test statistic (e.g. F, t, r) with confidence intervals, effect sizes, degrees of freedom and P value noted<br>*Give P values as exact values whenever suitable.*
☒	☐	For Bayesian analysis, information on the choice of priors and Markov chain Monte Carlo settings
☒	☐	For hierarchical and complex designs, identification of the appropriate level for tests and full reporting of outcomes
☒	☐	Estimates of effect sizes (e.g. Cohen's d, Pearson's r), indicating how they were calculated

Our web collection on statistics for biologists contains articles on many of the points above.

Software and code

Policy information about availability of computer code

Data collection	No software was used.
Data analysis	Microsoft Excel 2016 (version 16.0.5188.1000). GraphPad Prism (version 9.1.0). CLC Genomics Workbench 20.

For manuscripts utilizing custom algorithms or software that are central to the research but not yet described in published literature, software must be made available to editors and reviewers. We strongly encourage code deposition in a community repository (e.g. GitHub). See the Nature Portfolio guidelines for submitting code & software for further information.

Data

Policy information about availability of data

All manuscripts must include a data availability statement. This statement should provide the following information, where applicable:

- Accession codes, unique identifiers, or web links for publicly available datasets
- A description of any restrictions on data availability
- For clinical datasets or third party data, please ensure that the statement adheres to our policy

All transcriptomics data in this study are deposited in the European Nucleotide Archive (ENA), accession code ERP160633.

Field-specific reporting

Life sciences study design

All studies must disclose on these points even when the disclosure is negative.

Sample size	Culture samples (growth experiments and transcriptomics) were chosen to be performed with two or three biological replicates per condition (independent clones grown in identical conditions), as this allowed us to consider biological variance and still fit all assayed samples in well plates, as opposed to growing the same clone in independent cultures, which would not account for biological variance. Growth of samples on plates show a sample size of one, but was replicated with at least three independent experiments before making a conclusion. Competition assays were completed in technical duplicates but were replicated twice. For metabolomics, a sample size of four biological replicates (independent clones) was applied per condition, limited by analytical availability. In vitro sampling was performed with two technical replicates (identical reactions prepared in different samples), and here sample size was limited to two by the number of assay wells (8 total) and the inclusion of negative and positive controls.
Data exclusions	Only for in vivo experiments were data exclusions considered. As some of the strains were prone to mutagenesis, we pre-established through multiple replicated experiments the expected growth pattern of the strains applied. If any replicates deviated from this pattern, and were shown by following analysis to be mutated, the experiment was replicated.
Replication	All attempts at replication were successful. All experiments were replicated at least twice, with the only exception being transcriptomics, which was completed once with independent biological triplicates. For plate assays, color development was seen to be sensitive to even minor changes in timing and environment, and for these extra replications of experiments were completed to verify reproducibility.
Randomization	Random clones for each strain were selected for treatment. Randomization between strains was not completed, as to keep the possibility of monitoring the strains during experiments, as some were more prone to mutagenesis than others.
Blinding	Blinding was not considered as there were no subjective parameters or conscious subjects involved.

Reporting for specific materials, systems and methods

We require information from authors about some types of materials, experimental systems and methods used in many studies. Here, indicate whether each material, system or method listed is relevant to your study. If you are not sure if a list item applies to your research, read the appropriate section before selecting a response.

Materials & experimental systems

n/a	Involved in the study	
☐	☒	Antibodies
☒	☐	Eukaryotic cell lines
☒	☐	Palaeontology and archaeology
☒	☐	Animals and other organisms
☒	☐	Human research participants
☒	☐	Clinical data
☒	☐	Dual use research of concern

Methods

n/a	Involved in the study	
☒	☐	ChIP-seq
☒	☐	Flow cytometry
☒	☐	MRI-based neuroimaging

Antibodies

Antibodies used	For western blot, primary and secondary antibodies were applied to detect the target protein. These were anti-Crp (BioLegend, San Diego, CA, USA, Cat#664304, clone#1D8D9), anti-Lep (gifted from Stockholm University, Cat# unknown), anti-mouse (Sigma-Aldrich, Cat#AP124P, polyclonal) and anti-rabbit (Sigma Aldrich, Cat# unknown).
Validation	For validation of the Crp primary antibody, positive and negative controls were applied. This showed a band for protein in the case of WT E. coli (in Source Data), but not in the case of E. coli Delta-crp (Supplementary Fig. 2), thus validating the Crp antibody. The Lep primary antibody was validated in that it showed Lep as present in all strains as expected.
